# Complete Genome Sequences of Two *Lysobacter* Strains, Isolated from Seawater (*Lysobacter caseinilyticus*) and Soil (*Lysobacter helvus*) in South Korea

**DOI:** 10.1128/MRA.00337-21

**Published:** 2021-07-15

**Authors:** Yasuha Watanabe, Kazuharu Arakawa

**Affiliations:** a Japan Advanced Institute of Science and Technology, Nomi, Ishikawa, Japan; b Institute for Advanced Biosciences, Keio University, Tsuruoka, Yamagata, Japan; c Faculty of Environment and Information Studies, Keio University, Fujisawa, Kanagawa, Japan; d Systems Biology Program, Graduate School of Media and Governance, Keio University, Fujisawa, Kanagawa, Japan; e Exploratory Research Center on Life and Living Systems, National Institutes of Natural Sciences, Myodaiji, Okazaki, Japan; Montana State University

## Abstract

*Lysobacter* species produce lysobactin, a depsipeptide antibiotic that is effective against methicillin-resistant Staphylococcus aureus and vancomycin-resistant Staphylococcus aureus. Here, we report complete genome sequences of two *Lysobacter* strains, which were isolated from seawater (Lysobacter caseinilyticus) and soil (Lysobacter helvus) in South Korea.

## ANNOUNCEMENT

Members of the genus *Lysobacter* are Gram stain-negative, aerobic, rod-shaped bacteria classified in the phylum *Proteobacteria*, class *Gammaproteobacteria*, order *Xanthomonadales*, and family *Xanthomonadaceae*. *Lysobacter* species produce a characteristic antibiotic cyclic peptide known as lysobactin (1, 2), which is effective against methicillin-resistant Staphylococcus aureus (MRSA) and vancomycin-resistant Staphylococcus aureus (VRSA) (2). Therefore, to facilitate the understanding of these genes, we report here the complete genome sequences of two recently isolated species from South Korea, namely, Lysobacter caseinilyticus, which was isolated from seawater from Busan Harbor (3), and Lysobacter helvus, which was isolated from soil from the Dong-angyeong cave, Udo Island, Jeju, South Korea (4).

The two *Lysobacter* strains were obtained from RIKEN BioResource Research Center Microbe Division in freeze-dried form and were cultured in R2A liquid medium at 30°C for 2 days (L. caseinilyticus) or 1 week (L. helvus). Genome extraction and purification were carried out using the Genomic-tip 20/G kit (Qiagen). Preparation of the long-read sequence library was performed using a rapid barcoding kit (SQK-RBK004; Oxford Nanopore Technologies), and the library was sequenced in a FLO-MIN106 flow cell on the GridION device (Oxford Nanopore Technologies).The library for Illumina sequencing for error correction was prepared using a KAPA HyperPlus kit (Kapa Biosystems), and the library was sequenced on a NextSeq 500 sequencer (Illumina) using the 75-cycle high-output mode. The long-read sequencing produced a total yield of 64,316 reads (*N*_50_, 15.8 kbp) and 552,266 reads (*N*_50_, 14.7 kbp) for L. caseinilyticus and L. helvus, respectively. The assembly was performed using Canu v.2.11.0 (5) with reads filtered for length over 25 kbp (398 Mbp in total) and over 39 kbp (414 Mbp in total) for L. caseinilyticus and L. helvus, respectively, aiming for 100× coverage. The resulting single contig was manually circularized by deleting the overlapping ends. Totals of 2.7 million (L. caseinilyticus) and 2.9 million (L. helvus) unfiltered Illumina reads were mapped to the assembled genomes with Burrows-Wheeler Aligner (BWA) v.0.7.11 (6). The mapped sequence was subjected to hybrid error correction by Pilon v.1.24 polishing (7). Genome completeness was evaluated with the program CheckM (8) through the DDBJ Fast Annotation and Submission Tool (DFAST) pipeline (9), resulting in completeness of 100.0% for both genomes (taxonomy used: family *Xanthomonadaceae*). Annotation of the genome sequence was also performed using DFAST (9). Default parameters were used to run the software unless otherwise specified.

The annotated genome of L. caseinilyticus is 3,270,651 bp with a GC content of 67.9%, including 3,153 coding sequences (CDSs), 54 tRNAs, and 6 rRNAs. The annotated genome of L. helvus is 2,923,280 bp with a GC content of 69.4%, including 2,756 CDSs, 53 tRNAs, and 6 rRNAs. According to the annotation results, L. caseinilyticus and L. helvus both contain genes related to the lysobactin biosynthetic gene cluster (LybA and LybB) (10). When the lysobactin biosynthetic gene cluster was searched for using BLASTp (11), L. caseinilyticus showed high sequence similarity in LOCUS_04180, LOCUS_14750, and LOCUS_18050 and L. helvus in LOCUS_04180, LOCUS_14750, and LOCUS_18050 (E values of <1e−20).

### Data availability.

The genome sequences reported here were deposited in DDBJ under accession numbers AP024545 and AP024546, and the raw reads were deposited in the Sequence Read Archive (SRA) under BioProject accession numbers PRJNA716779 and PRJNA716799.
